# Identfication of Potent LXRβ-Selective Agonists without LXRα Activation by In Silico Approaches

**DOI:** 10.3390/molecules23061349

**Published:** 2018-06-04

**Authors:** Meimei Chen, Fafu Yang, Jie Kang, Huijuan Gan, Xuemei Yang, Xinmei Lai, Yuxing Gao

**Affiliations:** 1 College of Traditional Chinese Medicine, Fujian University of Traditional Chinese Medicine, Fuzhou 350122, China; meinuohua2007@163.com (J.K.); ghjzyz@163.com (H.G.); mei_tcm@163.com (X.Y.); keerer1990@163.com (X.L.); 2 College of Chemistry and Materials Science, Fujian Normal University, Fuzhou 350007, China; 3 College of Chemistry and Chemical Engineering, Xiamen University, Xiamen 361005, China; gaoxingchem@xmu.edu.cn

**Keywords:** LXRβ-selective agonists, QSAR modeling, molecular docking, Kohonen

## Abstract

Activating Liver X receptors (LXRs) represents a promising therapeutic option for dyslipidemia. However, activating LXRα may cause undesired lipogenic effects. Discovery of highly LXRβ-selective agonists without LXRα activation were indispensable for dyslipidemia. In this study, in silico approaches were applied to develop highly potent LXRβ-selective agonists based on a series of newly reported 3-(4-(2-propylphenoxy)butyl)imidazolidine-2,4-dione-based LXRα/β dual agonists. Initially, Kohonen and stepwise multiple linear regression SW-MLR were performed to construct models for LXRβ agonists and LXRα agonists based on the structural characteristics of LXRα/β dual agonists, respectively. The obtained LXRβ agonist model gave a good predictive ability (R^2^_train_ = 0.837, R^2^_test_ = 0.843, Q^2^_LOO_ = 0.715), and the LXRα agonist model produced even better predictive ability (R^2^_train_ = 0.968, R^2^_test_ = 0.914, Q^2^_LOO_ = 0.895). Also, the two QSAR models were independent and can well distinguish LXRβ and LXRα activity. Then, compounds in the ZINC database met the lower limit of structural similarity of 0.7, compared to the 3-(4-(2-propylphenoxy)butyl)imidazolidine-2,4-dione scaffold subjected to our QSAR models, which resulted in the discovery of ZINC55084484 with an LXRβ prediction value of pEC_50_ equal to 7.343 and LXRα prediction value of pEC_50_ equal to −1.901. Consequently, nine newly designed compounds were proposed as highly LXRβ-selective agonists based on ZINC55084484 and molecular docking, of which LXRβ prediction values almost exceeded 8 and LXRα prediction values were below 0.

## 1. Introduction

Numerous studies have demonstrated that high levels of plasma cholesterol induce dyslipidemia, atherosclerosis, and coronary heart diseases [1]. Liver X receptors (LXRs) are cholesterol sensors that protect cells from cholesterol overload [2]. Activating LXRs can stimulate reverse cholesterol transport from cells and inhibit its absorption and synthesis and promote HDL formation [3]. Currently, LXRs were identified as promising therapeutic targets for dyslipidemia, atherosclerosis, and cardiovascular diseases [4,5]. LXRs have two subtypes, including LXRα and LXRβ. LXRα is mainly expressed in liver, adipose tissue, intestine, and macrophages, and LXRβ is widely expressed in tissues. Both subtypes share approximately 78% homology in their ligand binding domains [6]. Activating LXRα results in undesired lipogenic effects such as increased hepatic lipogenesis, hypertriglyceridemia and liver steatosis [7], but growing evidence suggests that LXRβ-selective agonists can reduce these side effects [8,9]. Therefore, strategies to overcome the side effects related to LXRα activation in the treatment of dyslipidemia are to develop LXRβ-selective agonists, avoiding hepatic lipogenesis and the development of steatosis. However, given the little differences in their ligand binding domains, it can be difficult to obtain LXRβ-selective agonists without activation of LXRα.

To date, several computational approaches were also tried to predict LXR agonists. For instance, Susanne von Grafenstein, et al. identified novel LXR activators by structure-based modeling [10]. Also, Yali Li, et al. established QSAR classification models to distinguish between selective and non-selective LXRβ agonists by use of classification methods [11]. He peng, et al. identified the privileged chemical fragments of LXRβ agonists by application of a de novo substructure generation algorithm [12]. Veronika Temml, et al. discovered new LXR agonists by pharmacophore modeling and shape-based virtual screening [13]. However, these models did not make full use of the dual features of LXRs agonists, and did not judge whether these compounds were together with LXRα activity. Due to a high degree of similarity in the ligand binding domains of LXRβ and LXRα, it is very important to consider the LXRα activation when designing LXRβ agonists.

Therefore, the aim of this study was to apply a virtual screening workflow to simultaneously establish models for LXRβ and LXRα agonists as a fast filter to find highly potent LXRβ-selective agonists without LXRα activation, based on a series of new reported 3-(4-(2-propylphenoxy)butyl)imidazolidine-2,4-dione-based LXRα/β dual agonists, and also mined the structural features responsible for their selective activity of LXRα/β. Firstly, a Kohonen’s self-organizing map and multiple linear regression combined with a stepwise technology were performed to construct models for LXRβ agonists and LXRα agonists based on the same scaffolds of LXRα/β dual agonists, respectively. Secondly, the compounds in the ZINC database that fulfilled the requirement of a structural similarity of 0.7 compared to known reported LXRs agonists were subjected to the two QSAR models to screen for new LXRβ-selective agonists. Then, to discover highly potent LXRβ-selective agonists without LXRα activation, two QSAR models were further applied to design new compounds based on above screened compounds. Finally, molecular docking was applied to understand their binding interactions in the LXRβ binding site.

## 2. Results and Discussion

### 2.1. Results of Dataset Division by Kohonen Map 

**Figure 1:**
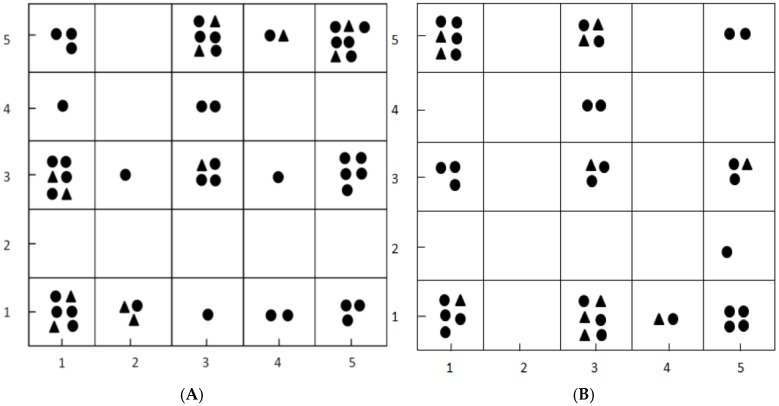
The Kohonen maps for the training set and test set for LXRβ modeling (**A**) and LXRα modeling (**B**): black dots represent compounds of the training set and black triangles represent compounds of the test set.

After the descriptors reduction, totally 183 molecular descriptors were used as variables to build Kohonen maps (5 × 5 neurons, 500 epochs). On the basis of the trained network, the compounds fell into different neuron of the Kohonen map (see Figure 1). Similar chemicals were within the same cell of the Kohonen map. The selection of the training set chemicals was performed by the minimal distance from the centroid of each cell in the Kohonen map [14]. The remaining objects, close to the training set chemicals, were used as the test set. As a result, there were 40 compounds in the training set and 12 compounds in the test set used for building models for LXRβ agonists, additionally, 30 compounds in the training set and 11 compounds in the test set used for constructing models for LXRα agonists. Figure 1 showed the distribution of the training set and test set, marked by circle and triangle symbols, respectively.

### 2.2. MLR Model Results of LXRβ Activity

After SW-MLR was performed, the best QSAR model for LXRβ agonists was generated with nine molecular descriptors. The obtained QSAR model was shown as follows:

pEC_50_ = −0.777 × vsurf_IW2 + 0.007 × SMR_VSA6 − 1.236 × glob + 4.560 × GCUT_SLOGP_2 − 60.185 × E_strain − 0.189 × dipoleX − 0.247 × AM1_LUMO + 0.154 × vsurf_IW5 + 0.016 × vsurf_DD13 + 5.087

N_train_ = 40, R^2^_train_ = 0.837, F_train_ = 17.235 > F_0.01_ (9, 30) = 3.06 (the cut off value of F distribution), RMSE_train_ = 0.118, Q^2^_LOO_ = 0.715, RMSE _LOO_ = 0.156, N_test_ = 12, R^2^_test_ = 0.843, RMSE_test_ = 0.534.

**Figure 2:**
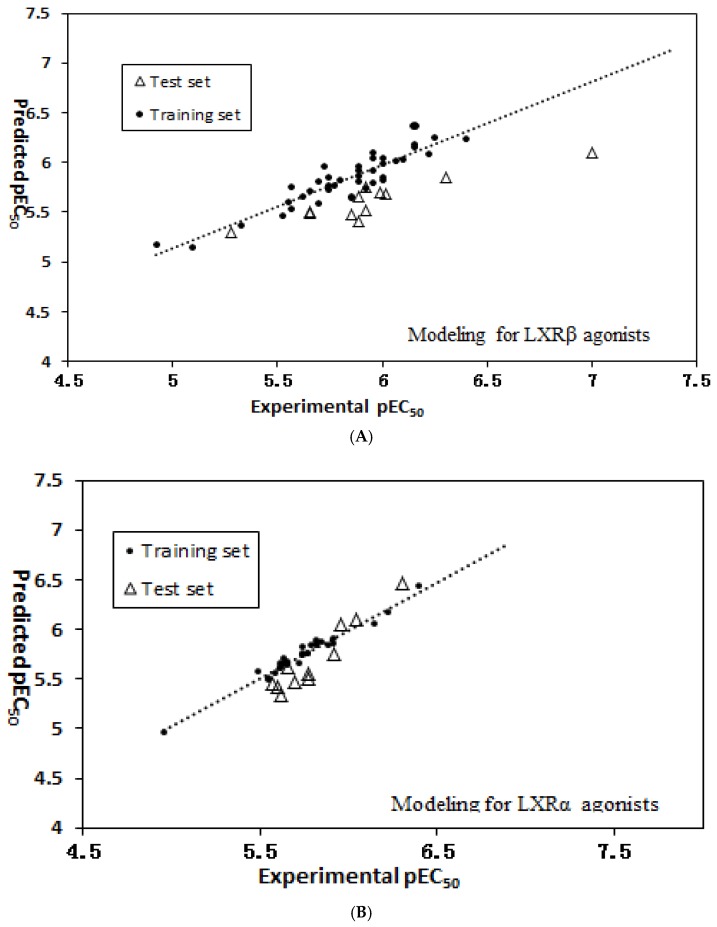
Plots of experimental pEC_50_ values against predicted pEC_50_ values by QSAR models for LXRβ agonists (**A**) and LXRα agonists (**B**).

The selected variables and their chemical meanings, standard coefficients, significance and variable inflation factor (VIF) were presented in Table 1. It can be seen that all selected descriptors had a statistical significance of less than 0.05, indicating that they were obvious features in defining the activity of LXRβ-selective agonists. As shown in Table 1, the VIF of all descriptors was smaller than 5, indicating no multicollinearity existed among the descriptors in models [15]. Table S2 (see Supplementary Materials) lists the correlation matrix of the selected descriptors in the QSAR model. All linear correlation coefficient values for each pair of descriptors were smaller than 0.85, showing that they were independent [16]. The predicted results of QSAR model are given in Table S1 (see Supplementary Materials) and shown in Figure 2. As described in Table 2, obviously, the QSAR model was very successfully built with statistical significance and good prediction ability. The R^2^_train_ value of this model reveals that it can explain 83.7% of variance in activity. The Q^2^_LOO_ value of 0.715 was bigger than 0.5, indicating that the developed model had very good stability and predictive ability. In addition, the value of R^2^_test_ for the external prediction was 0.843, showing good prediction and generalization ability.

### 2.3. QSAR Model Results of LXRα Activity

After the SW-MLR was performed, the best QSAR model for LXRα activators was generated with twelve molecular descriptors. The obtained QSAR model was generated as follows: 

The selected variables and their chemical meanings, standard coefficients, and variable inflation factors are shown in Table 3. The values of VIF and significance showed that these 12 descriptors were obvious features in defining LXRα activity. All linear correlation coefficient values for each pair of descriptors were smaller than 0.85, showing that they were independent [16]. The predicted results of the QSAR model were listed in Table S3 (see Supplementary Materials) and shown in Figure 2. Table 2 listed the statistical results of the proposed model. As described in Table 2, the obtained QSAR model was very successful and of good predictive ability. The QSAR model can give 96.8% variances in LXRα activity in the training set. The Q^2^_LOO_ value of leave-one-out (LOO) cross-validation was 0.895 (much bigger than 0.5), showing that the developed QSAR model had good stability and predictive ability. Additionally, the R^2^_test_ for the external prediction also reached 0.914, indicating good prediction and generalization ability of the LXRα QSAR model [17].

Finally, the Y-randomization tests were performed to confirm the robustness of two QSAR models [18]. Table 4 listed the results of ten Y-randomization tests for these two LXRα and LXRβ QSAR models. It can be observed that all new R^2^_train_ and Q^2^_LOO_ values of the Y-randomization tests were much smaller than those of the original models. Thereby, the two QSAR models with good predictive abilities were not due to a chance correlation or structural dependency of the training set. Overall, these two QSAR models for LXRα and LXRβ agonists were quite satisfied, exhibiting the significantly high predictive ability, reliability, and robustness, which can be used to predict LXRβ and LXRα activity.

### 2.4. Interpretation of the Descriptors

It is possible to gain some vital structural features to govern the LXRβ-selective activity by interpreting the molecular descriptors in the QSAR models. In the QSAR models of LXRβ agonists and LXRα agonists, nine and twelve descriptors were uncovered, respectively. Additionally, only one descriptor (GCUT_SLOGP_2) is the same for two QSAR models. In order to investigate whether there was some correlation between two QSAR models, the correlation coefficients of their descriptors were calculated as listed in Table 5. Obviously, all linear correlation coefficient values for each pair of descriptors between two QSAR models were smaller than 0.6, indicating that these two QSAR models were independent and can well distinguish LXRβ and LXRα activity.

### 2.5. Screening New Highly LXRβ-Selective Agonists

In general, compounds with high structural similarity (bigger than 0.7) to the basic scaffold of the training set will be given more accurate predictions than compounds without similarity [19], and will also have similar biological activity [20]. Thus, to ensure maximum accuracy of these predictions, the structural similarity between compounds in the ZINC database and 3-(4-phenoxybutyl)imidazolidine-2,4-dione skeleton were calculated. Compounds with structural similarity below 0.7 were removed from the ZINC database, which resulted in the retrieval of 637 compounds. The compounds with predictive activity values (pEC_50_ > 6.0 for LXRβ and pEC_50_ < 1.0 for LXRα) were identified as potential LXRβ-selective agonists. Thus, 11 compounds were discovered from these molecules based on our QSAR models. Among them, ZINC55084484 had the best LXRβ prediction values (pEC_50_ = 7.343) and LXRα prediction values (pEC_50_ = −1.901), much better in LXRβ-selective activity than the best reported compounds (pEC_50_ = 7.0 for LXRβ and pEC_50_ = 6.095 for LXRα) in Table S1 (see Supplementary Materials). Thus, to find highly potent LXRβ-selective agonists, we designed new compounds based on ZINC55084484. As listed in Table 6, we found that the presence of absorbent groups such as propionyloxy, propionamido and 2,2,2-trifluoroethylamino at R1 and R3 of the benzene ring can significantly enhance LXRβ agonist activity, among which the addition of 2,2,2-trifluoroethylamino at R1 (Table 6) performed best. It was also observed that the addition of a chlorine atom at the ortho or para position of 2,2,2-trifluoroethylamino can lead to better LXRβ agonist activity, such as compounds N1 and N3 with predicted pEC_50_ values of 8.497 and 8.429, respectively.

### 2.6. Molecular Docking Study

**Figure 3:**
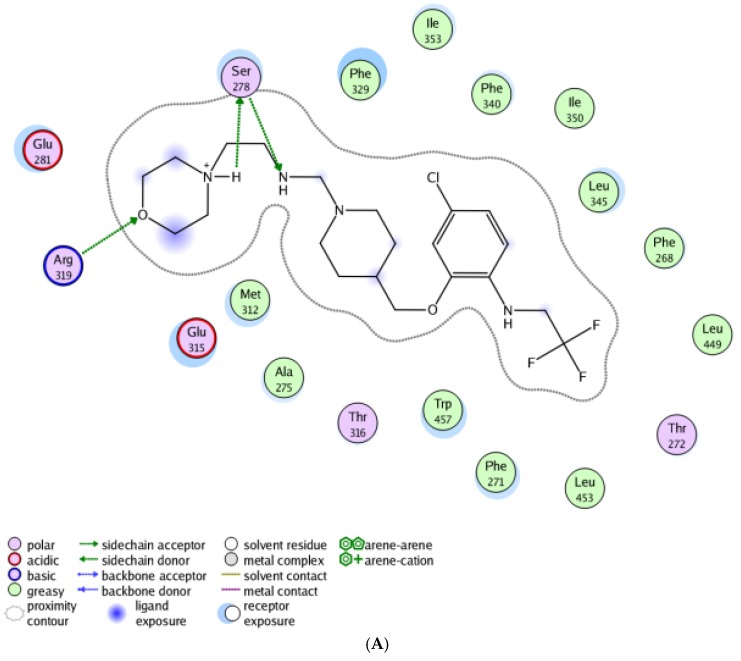

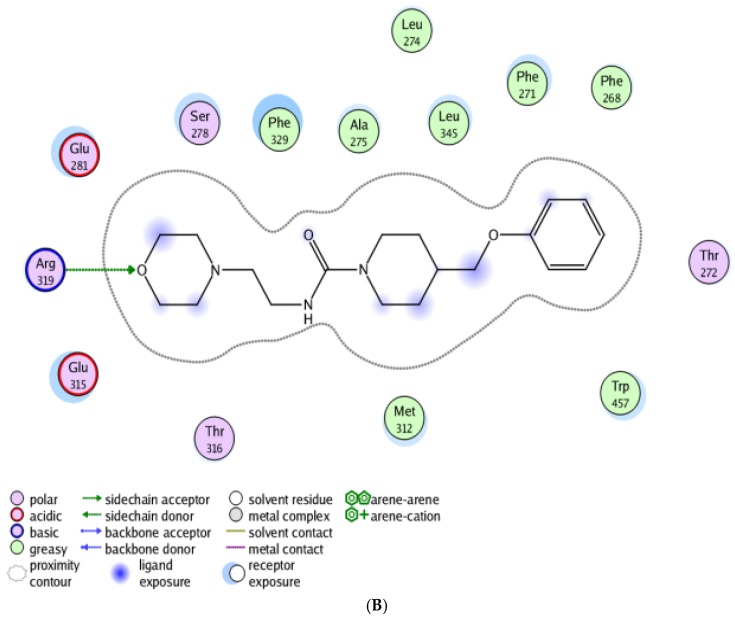
The binding modes of new designed compound N1 (**A**) and the template compound (ZINC55084484) (**B**) in the LXRβ active site.

Molecular docking embedded in Molecular Operating Environment software (MOE2008.10, Chemical Computing Group Inc., Montreal, QC, Canada) was applied to better understand the binding modes and important interactions of new designed LXRβ-selective agonists. In this docking study, the root-mean-square distance (RMSD) parameter of the ligand between the three-dimensional crystal structure of the LXRβ complex (PDB: 5JY3) and in the redocked structure was 0.807 Å, showing that these docking parameters were suitable, and the docking results were reliable [21]. Docking results of the newly designed LXRβ-selective agonists were listed in Table 6. Obviously, these newly designed compounds had better docking scores for LXRβ than the template compound (ZINC55084484), which were in agreement with the QSAR results. It was also observed that the presence of absorbent groups such as propionyloxy and 2,2,2-trifluoroethylamino at R^1^ and R^3^ of benzene ring significantly enhanced the LXRβ agonist activity, which almost corresponded with the QSAR results. The best docked conformation of the most active compound N1, as shown in Figure 3, revealed that the presence of 2,2,2-trifluoroethylaminoand chlorine at R^1^ and R^3^ of benzene ring allowed for potentiation of strong hydrophilic interactions with Phe340, Ile345, Phe268, Ala343, Phe268, Leu449, Thr272, Leu453, Phe271 and Trp457 in the active site of LXRβ. Comparative to molecular docking between compound N1 and the template compound ZINC55084484, shown in Figure 3, the former had a better binding score than the latter. This revealed that carbonyl was not conducive to the activity compared with methylene in the X place, limiting the molecular flexibility, and the presence of 2,2,2-trifluoroethylamino and chlorine at R^1^ and R^3^ of benzene ring allowed for potentiation of the strong hydrophilic interactions in the active site of LXRβ. It can be concluded that more H-bonds and hydrophobic interactions between substituent groups at benzene ring with above amino acids were beneficial to the activity.

Additionally, a drug-likeness analysis was also performed to evaluate the oral drug-like property of these new designed LXRβ-selective agonists using the Lipinski rule of five, which predicts that great absorption or permeation is more likely when molecular weight is no more than 500 Da, the number of H-bond donors (a don) are less than five, the number of H-bond acceptors (a_acc) are less than 10 and the octanol-water partition coefficient (logP(o/w)) is lower than five [19,22]. The drug-like property descriptors of these compounds were listed in Table 7. All values of five such descriptors were largely coincided with the five rules of oral medications. Thereby, these nine newly designed compounds were suggested to be highly LXRβ-selective agonists.

## 3. Materials and Methods

### 3.1. Dataset Division

**Figure 4:**
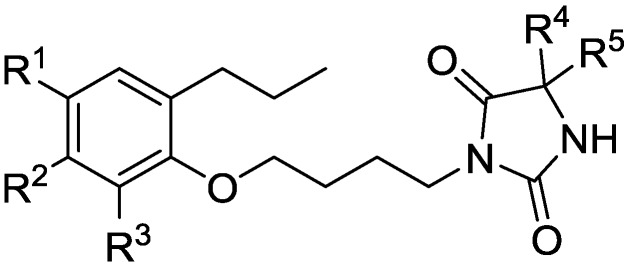
3-(4-(2-propylphenoxy)butyl)imidazolidine-2,4-dione based LXRα/β dual agonists.

In this QSAR analysis, a series of fifty-three 3-(4-(2-propylphenoxy)butyl)imidazolidine-2,4-dione-based LXRα/β dual agonists were taken from K. Shibuya [23,24] and the basic scaffold was presented in Figure 4. The activated activity (EC_50_) values were covered to logarithmic scale pEC_50_ values, which were used as the dependent parameters in the QSAR study. The molecular structures and activity data of LXRs agonists were presented in Table S1 (see Supplementary Materials). All 2D structures of compounds in Table S1 were sketched using ChemDraw software and were converted into 3D structures using energy minimization module embedded in Molecular Operating Environment software (MOE2008.10, Chemical Computing Group Inc., Montreal, QC, Canada). Then, their conformer structures were optimized by stochastic conformational search and followed to generate 327 diverse descriptors by utilizing the QSAR module of MOE [25]. The redundant information among descriptors was conducted by deleting constant or almost constant values for all molecules and removing one of inter-correlated descriptors (a pairwise correlation coefficient greater than 0.95) [26]. Finally, a total set of 183 descriptors remained and were used for QSAR modeling. To obtain reliable QSAR models, the studied chemicals were firstly separated into a training set and a test set using a Kohonen’s self-organizing map (5 × 5 neurons, 500 epochs), which ensured the training set spanned the whole descriptor space and kept a balanced distribution of the chemicals in two data sets [27]. 

### 3.2. Stepwise Multiple Linear Regression (SW-MLR) 

Feature selection is considered as one of the key steps in development of 2D-QSAR models. In this study, a stepwise technology combined with MLR (SW-MLR) was applied to select a suitable set of descriptors that could be used as input values for model construction. The stepwise regression is a variation on forward selection. At each stage in the process, after adding a new variable, an F-test was performed to check if some variables could be removed without significantly increasing the residual sum of squares [28]. So, different MLR models were developed in this procedure. The statistical parameters such as squared correlation coefficient (R^2^), root mean standard error (RMSE), and Fisher statistic were calculated to assess the performance of derived QSAR models [29].

### 3.3. Model Validation

Model validation is a critical step in assessing the predictive ability and reliability of QSAR models. It includes internal and external validations. Generally, the leave-one-out (LOO) cross-validation technology is often considered as the most economical and popular internal validation to evaluate the predictive ability of the model [30]. LOO cross-validation involves using one object from the dataset as the validation set, and the remaining dataset serves as the training data. This is repeated so that every object in the dataset is used once as the validation data, which employs all the information available. Usually, the model is acceptable when the value of LOO cross-validation squared correlation coefficient (Q^2^_LOO_) is bigger than 0.5 [13]. Moreover, external validation is significant and essential to evaluate the generalization performance of the proposed model. The statistical parameters, such as the squared correlation coefficient (R^2^_test_) and root mean square errors (RMSE_test_) of the test set were calculated to evaluate the performance of the model [29].

All algorithms were written in MATLAB 8.0 and run on a computer [Intel(R) Pentium(R), 2.00-GB RA].

### 3.4. Screening News LXRβ-Selective Agonists

The ZINC database that contained over 35 million diverse purchasable compounds was subjected to our QSAR model prediction for discovering new highly-potent LXRβ-selective agonists [31]. Given that our QSAR models were constructed based on a 3-(4-phenoxybutyl)imidazolidine-2,4-dione skeleton, only compounds with these skeletons can be well-predicted by our QSAR models. Thereby, the molecular structural similarity between compounds in ZINC database and 3-(4-phenoxybutyl)imidazolidine-2,4-dione skeleton was first calculated using the Tanimoto coefficient in Open Bable 2.3.1 [32]. Generally, a good cutoff for the Tanimoto coefficient for biologically similar molecules is 0.7 or 0.8 [20]. Here, compounds with a structural similarity bigger than 0.7 were selected out from the ZINC database and imported into MOE for further analysis. Hydrogen atoms and partial charges were assigned, and then they were energy minimized using the molecular mechanics force field method with a convergence criterion of 0.01 kcal/mol. Then, the two above obtained QSAR models were applied to screen new LXRβ-selective agonists from these compounds. Subsequently, to discover highly potent LXRβ-selective agonists that do not activate LXRα, the models were further applied to design new compounds based on above screened compounds from ZINC database.

### 3.5. Molecular Docking Study 

Molecular docking was further performed to study the binding modes and important interactions of new designed LXRβ-selective agonists. The docking simulation was carried out as follows [33]. First, the three-dimensional crystal structure of the LXRβ-GW3965 complex from the RSCB protein databank (PDB: 5JY3) was protonated using AMBER99 force field and minimized with a RMSD gradient of 0.05 kcal/mol Å. In addition, the binding site and docking placement were using the ligand atom mode and trianglematcher algorithm, respectively. Finally, two rescoring methods including London dG and Affinity dG, along with the force field method, were adopted to compute the interactions.

## 4. Conclusions

In this paper, modeling techniques such as Kohonen and SW-MLR, structural similarity analysis, and molecular docking were successfully applied to establish models to develop highly potent LXRβ-selective agonists without activation of LXRα based on a series of newly reported LXRα/β dual agonists. The best obtained QSAR model for LXRβ can explain 83.7% of the variance in activity with a low RMSE of 0.118, and the best derived QSAR model for LXRα can give better predictive ability with R^2^_train_ of 0.968 and RMSE of 0.045. Also, the two QSAR models uncovered approximately different important features in defining LXRα and LXRβ activity. They were independent and could well distinguish LXRβ and LXRα activity. A total of 11 compounds from the ZINC database that fulfilled the requirement of structural similarity of 0.7 compared to known dual LXRα/β agonists were predicted with activity values of pEC_50_ > 6.0 for LXRβ and pEC_50_ < 1.0 for LXRα. Among them, ZINC55084484 had the best LXRβ prediction values (pEC_50_ = 7.343) and LXRα prediction value (pEC_50_ = −1.901), much better in LXRβ-selective activity than the best reported compounds in Table S1 (pEC_50_ = 7.0 for LXRβ and pEC_50_ = 6.095 for LXRα). Thereupon, nine new compounds were designed as highly potent LXRβ-selective agonists based on ZINC55084484, of which LXRβ prediction values almost surpassed 8 and LXRα prediction values were below 0. Additionally, the docking results of the newly designed LXRβ-selective agonists corresponded with the QSAR results well. The best docked conformation of the most active compound N1 revealed that carbonyl was not conducive to the activity compared with methylene in the X place, limiting the molecular flexibility, and the presence of 2,2,2-trifluoroethylamino and chlorine at R^1^ and R^3^ of the benzene ring allowed for potentiation of strong hydrophilic interactions in the active site of LXRβ. Overall, this study could provide valuable guidance for the future design of highly potent LXRβ-selective agonists in the drug discovery process.

## Figures and Tables

**Table 1:** Selected descriptors of MLR model for LXRβ agonists.

Descriptor	Chemical Meaning	Coefficient	Standard Coefficient	VIF	*p*-Value
vsurf_IW2	Hydrophilic integy moment	−0.777	−0.592	2.246	0.000
SMR_VSA6	Sum of vi such that Ri is in (0.485,0.56]	0.007	0.325	1.232	0.000
glob	Globularity, or inverse condition number (smallest eigenvalue divided by the largest eigenvalue) of the covariance matrix of atomic coordinates.	−1.236	−0.668	1.779	0.000
GCUT_SLOGP_2	The GCUT descriptors using atomic contribution to logP	4.560	0.687	2.785	0.000
E_strain	Local strain energy	−60.185	−0.315	1.196	0.000
dipoleX	The *x* component of the dipole moment	−0.189	−0.358	1.240	0.000
AM1_LUMO	The energy (eV) of the Lowest Unoccupied Molecular Orbital calculated using the AM1 Hamiltonian	−0.247	−0.397	2.712	0.003
vsurf_IW5	Hydrophilic integy moment	0.154	0.284	1.790	0.007
vsurf_DD13	Contact distances of vsurf_DDmin	0.016	0.212	1.192	0.013
Constant		5.087			

**Table 2:** Statistical parameters of two QSAR models for LXRβ and LXRα agonists.

	Training Set					Test Set	
QSARModel	R^2^_train_	RMSE_train_	F	Q^2^_LOO_	RMSE_LOO_	R^2^_test_	RMSE_test_
LXR beta	0.837	0.118	17.235	0.715	0.156	0.843	0.232
LXR alpha	0.968	0.045	44.068	0.895	0.081	0.914	0.155

**Table 3:** Selected descriptors of MLR model for LXRα agonists.

Descriptor	Chemical Meaning	Coefficient	Standard Coefficient	VIF	*p*-Value
GCUT_SLOGP_2	The GCUT descriptors using atomic contribution to logP	8.952	1.539	4.862	0.000
vsurf_DD12	Contact distances of vsurf_DDmin	0.024	0.366	1.348	0.000
Q_VSA_POS	Total positive van der Waals surface area	0.005	0.673	2.161	0.000
SlogP_VSA2	Sum of vi such that Li is in (−0.2,0]	0.023	0.813	4.763	0.000
E_ang	Angle bend potential energy	−0.019	−0.325	2.126	0.000
pmiY	*y* component of the principal moment of inertia	4.808 × 10^−5^	0.209	1.468	0.001
dipoleY	The *y* component of the dipole moment	0.164	0.281	1.408	0.000
vsurf_DW12	Contact distances of vsurf_EWmin	−0.019	−0.203	1.467	0.001
BCUT_SMR_0	The BCUT descriptors using atomic contribution to molar refractivity	34.772	0.351	2.546	0.000
SlogP_VSA3	Sum of vi such that Li is in (0,0.1]	0.005	0.238	1.590	0.000
vsurf_CW6	Capacity factor	−4.271	−0.273	3.370	0.003
Q_VSA_FPPOS	Fractional positive polar van der Waals surface area	−1.339	−0.144	1.810	0.024
Constant		83.858			

**Table 4:** R^2^_train_ and Q^2^_LOO_ values of QSAR models after ten Y-randomization tests.

No. of Test		1	2	3	4	5	6	7	8	9	10
LXRβ model	*R*^2^train	0.185	0.182	0.155	0.257	0.282	0.208	0.126	0.244	0.155	0.241
	*Q* ^2^_LOO_	0.063	0.006	0.018	0.001	0.037	0.015	0.081	0.002	0.047	0.003
LXRα model	*R* ^2^_train_	0.259	0.174	0.288	0.287	0.334	0.298	0.25	0.264	0.258	0.287
	*Q* ^2^_LOO_	0.033	0.097	0.086	0.009	0.015	0.039	0.023	0.016	0.032	0.027

**Table 5:** Correlation coefficients of descriptors between two QSAR models.

	AM1_LUMO	GCUT_SLOGP_2	E_strain	dipoleX	SMR_VSA6	vsurf_DD13	vsurf_IW2	vsurf_IW5	Glob
BCUT_SMR_0	0.031	−0.255	0.254	0.199	−0.243	−0.172	0.075	−0.069	−0.063
GCUT_SLOGP_2	−0.055	0.014	0.066	−0.194	−0.371	0.090	0.226	−0.133	0.110
Q_VSA_FPPOS	−0.242	−0.315	0.072	−0.008	−0.041	0.258	−0.178	−0.184	−0.117
Q_VSA_POS	−0.017	0.279	−0.182	−0.164	0.240	0.113	−0.202	−0.012	0.013
E_ang	−0.069	−0.431	0.007	−0.030	0.173	0.531	0.148	−0.261	−0.361
dipoleY	−0.216	0.045	−0.168	−0.189	0.051	−0.001	−0.062	−0.086	0.254
pmiY	0.204	−0.159	−0.195	−0.098	−0.020	0.110	0.216	0.067	−0.353
SlogP_VSA2	0.029	−0.166	−0.012	0.083	0.210	0.054	−0.076	0.051	−0.347
SlogP_VSA3	−0.155	0.246	0.095	−0.032	−0.236	0.015	0.026	0.082	0.085
vsurf_CW6	−0.056	0.002	0.352	−0.231	−0.116	−0.127	−0.137	−0.156	0.201
vsurf_DD12	−0.564	0.005	0.161	0.067	−0.400	0.226	0.243	0.060	−0.211
vsurf_DW12	0.116	−0.075	−0.046	−0.324	0.099	−0.099	−0.092	−0.304	−0.091

**Table 6:** Chemical structures of newly designed LXRβ-selective agonists based on two QSAR Models.

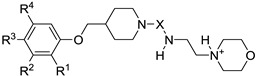								
Name	R^1^	R^2^	R^3^	R^4^	X	Predicted pEC_50_ Values		
						LXRβ	LXRα	Docking Scores
**ZINC55084484**	H	H	H	H	CO	7.343	−1.901	−7.713
**N1**	2,2,2-trifluoroethylamino	H	H	Cl	CH_2_	8.497	−1.911	−11.205
**N2**	2,2,2-trifluoroethylamino	H	H	H	CH_2_	8.390	−2.076	−9.394
**N3**	2,2,2-trifluoroethylamino	Cl	H	H	CH_2_	8.429	−1.730	−9.757
**N4**	2,2,2-trifluoroethylamino	F	H	H	CO	8.328	0.215	−10.236
**N5**	propionyloxy	H	H	H	CO	8.148	−0.753	−9.528
**N6**	2,2,2-trifluoroethylamino	H	propionyloxy	H	CH_2_	7.932	−0.9524	−9.323
**N7**	propionyloxy	H	propionyloxy	H	CO	7.923	−0.905	−9.177
**N8**	2,2,2-trifluoroethylamino	F	H	H	CH_2_	8.178	−1.760	−10.068
**N9**	2,2,2-trifluoroethylamino	H	propionyloxy	H	CO	8.111	−1.211	−10.321

**Table 7:** Drug-like property descriptors of new designed LXRβ-selective agonists.

Name	Predicted pEC_50_ Values		Weight	a_acc	a_don	logP(o/w)
	LXRβ	LXRα				
**ZINC55084484**	7.343	−1.901	347.459	4	1	1.218
**N1**	8.497	−1.911	465.968	4	2	2.757
**N2**	8.390	−2.076	431.523	4	2	2.128
**N3**	8.429	−1.730	465.968	4	2	2.718
**N4**	8.328	0.215	463.496	3	2	1.777
**N5**	8.148	−0.753	420.53	4	1	1.376
**N6**	7.932	−0.9524	503.586	5	2	2.573
**N7**	7.923	−0.905	492.593	5	1	1.821
**N8**	8.178	−1.760	449.513	4	2	2.279
**N9**	8.111	−1.211	517.569	4	2	2.071

## Supplementary Materials

The following are available online, Table S1: Molecular structures and corresponding pEC_50_ values of experimental and predicted of the 3-(4-(2-propylphenoxy)butyl)imidazolidine-2,4-dione based LXRα/β dual agonists, Table S2: The correlation matrix of descriptors of LXRβ QSAR model, Table S3: The correlation matrix of descriptors of LXRα QSAR model.
