# Supplementary material for: Identfication of Potent LXRβ-Selective Agonists without LXRα Activation by In Silico Approaches
Source: Molecules. 2018 Jun 4;23(6):1349. doi: 10.3390/molecules23061349 (PMC6099648; doi:10.3390/molecules23061349)
Supplement: Supplementary file 1 [file molecules-23-01349-s001.pdf]

Identfication of Potent LXRβ-Selective Agonists without LXRα Activation by In Silico Approaches

Meimei Chen [1,2,*], Fafu Yang [2,*], Jie Kang [1], Huijuan Gan [1], Xuemei Yang [1], Xinmei Lai [1] and Yuxing Gao [3]

Table S1. Molecular structures and corresponding pEC_{50} values of experimental and predicted of the 3-(4-(2-propylphenoxy)butyl)imidazolidine-2,4-dione based LXRα/β dual agonists.

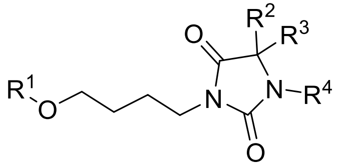

ID	R^1	R2	R3	R4	pEC_{50}^{α} (μM)	Predicted pEC_{50}^{α}	pEC_{50}^{β} (μM)	Predicted pEC_{50}^{β}
1	2-oxo-8-propyl-4-(trifluoromethyl)-2H-chromen-7-yl	CH_3	CH_3	CH_3	5.620	5.621	6.000	5.827
2	2-oxo-8-propyl-4-(trifluoromethyl)-2H-chromen-7-yl	CH_3	4-methoxy-phenyl	H	ia	ia	5.854	5.651
3	8-propyl-4-(trifluoromethyl)chroman-6-yl	CH_3	CH_3	CH_3	nd	nd	5.658	5.718
4^{β}	4-(1,1,1,3,3,3-hexafluoro-2-hydroxypropan-2-yl)-6-propylphenyl	CH_3	3,4-dimethoxy phenyl	H	5.620	5.654	5.886	5.654
5	4-propyl-1,1-bis(trifluoromethyl)-1,3-dihydroisobenzofuran-5-yl	CH_3	CH_3	CH_3	5.658	5.638	6.066	6.017
6^{α}	4-(1,1,1,3,3,3-hexafluoro-2-hydroxypropan-2-yl)-6-propylphenyl	CH_3	Phenyl	H	5.699	5.479	4.921	5.173
7	4-propyl-1,1-bis(trifluoromethyl)-1,3-dihydroisobenzofuran-5-yl	CH_3	4-methoxy-phenyl	H	5.770	5.750	6.000	6.040
8	4-(1,1,1,3,3,3-hexafluoro-2-hydroxypropan-2-yl)-6-propylphenyl	CH_3	CH_3	CH_3	5.495	5.570	5.721	5.960
9	3-(1,1,1,3,3,3-hexafluoro-2-hydroxypropan-2-yl)-5-propylphenyl	CH_3	4-methoxy-phenyl	H	5.553	5.488	5.745	5.730
10^{β}	4-(1,1,1,3,3,3-hexafluoro-2-hydroxypropan-2-yl)-6-propylphenyl	CH_3	3-methoxy-phenyl	H	5.620	5.599	5.658	5.4978
11	4-(1,1,1,3,3,3-hexafluoro-2-hydroxypropan-2-yl)-2,6-dipropylphenyl	CH_3	CH_3	CH_3	6.149	6.059	6.252	6.257
12	4-(1,1,1,3,3,3-hexafluoro-2-hydroxypropan-2-yl)-2,6-dipropylphenyl	CH_3	4-methoxy-phenyl	H	5.796	5.841	5.854	5.654

13[α]	4-(1,1,1,3,3,3-hexafluoro-2-hydroxypropan-2-yl)-6-propylphenyl	CH_3	4-methyl-phenyl	H	5.658	5.624	5.699	5.583
14[β]	4-(1,1,1,3,3,3-hexafluoro-2-hydroxypropan-2-yl)-6-propylphenyl	CH_3	4-isopropylphenyl	H	5.721	5.649	5.658	5.509
15	4-(1,1,1,3,3,3-hexafluoro-2-hydroxypropan-2-yl)-6-propylphenyl	CH_3	4-ethoxyphenyl	H	5.886	5.836	5.959	5.922
16	4-(1,1,1,3,3,3-hexafluoro-2-hydroxypropan-2-yl)-6-propylphenyl	CH_3	4-isopropoxyphenyl	H	5.745	5.761	5.553	5.606
17	4-(1,1,1,3,3,3-hexafluoro-2-hydroxypropan-2-yl)-6-propylphenyl	CH_3	4-(trifluoromethoxy)phenyl	H	5.553	5.501	5.921	5.737
18[β]	4-(1,1,1,3,3,3-hexafluoro-2-hydroxypropan-2-yl)-6-propylphenyl	CH_3	benzo[d][1,3]dioxol-5-yl	H	5.770	5.754	6.301	5.854
19[α]	4-(1,1,1,3,3,3-hexafluoro-2-hydroxypropan-2-yl)-6-propylphenyl	CH_3	2,3-dihydrobenzo[b][1,4]dioxin-6-yl	H	5.921	5.746	6.398	6.240
20	4-(1,1,1,3,3,3-hexafluoro-2-hydroxypropan-2-yl)-6-propylphenyl	CH_3	4-(dimethylamino)phenyl	H	5.658	5.678	5.569	5.528
21	4-(1,1,1,3,3,3-hexafluoro-2-hydroxypropan-2-yl)-6-propylphenyl	CH_3	4-chlorophenyl	H	5.658	5.648	5.745	5.772
22[α]	4-(1,1,1,3,3,3-hexafluoro-2-hydroxypropan-2-yl)-6-propylphenyl	CH_3	3,4-di chlorophenyl	H	5.770	5.556	5.959	6.050
23	4-(1,1,1,3,3,3-hexafluoro-2-hydroxypropan-2-yl)-6-propylphenyl	CH_3	1,1'-biphenyl-4-yl	H	5.854	5.875	6.000	5.851
24[α]	4-(1,1,1,3,3,3-hexafluoro-2-hydroxypropan-2-yl)-6-propylphenyl	CH_3	4-(trifluoromethyl)phenyl	H	5.602	5.429	5.745	5.849
25[β]	4-(1,1,1,3,3,3-hexafluoro-2-hydroxypropan-2-yl)-2,6-dipropylphenyl	CH_3	benzo[d][1,3]dioxol-5-yl	H	6.398	6.428	7.000	6.095
26	4-(1,1,1,3,3,3-hexafluoro-2-hydroxypropan-2-yl)-6-propylphenyl	ethyl	4-methoxyphenyl	H	5.824	5.883	6.000	5.986
27	4-(1,1,1,3,3,3-hexafluoro-2-hydroxypropan-2-yl)-6-propylphenyl	ethyl	benzo[d][1,3]dioxol-5-yl	H	5.638	5.656	6.155	6.156
28[α]	4-(1,1,1,3,3,3-hexafluoro-2-hydroxypropan-2-yl)-6-propylphenyl	CH_3	2-methoxy-phenyl	H	5.569	5.453	ia	ia
29	4-(1,1,1,3,3,3-hexafluoro-2-hydroxypropan-2-yl)-6-propylphenyl	propyl	benzo[d][1,3]dioxol-5-yl	H	5.824	5.839	5.886	5.916
30	4-(1,1,1,3,3,3-hexafluoro-2-hydroxypropan-2-yl)-6-propylphenyl	ethyl	2,3-dihydrobenzo[b][1,4]dioxin-6-yl	H	5.638	5.712	6.155	6.368
31[α]	4-(1,1,1,3,3,3-hexafluoro-2-hydroxypropan-2-yl)-2,6-dipropylphenyl	CH_3	4-ethoxyphenyl	H	6.046	6.097	6.097	6.036
32[β,α]	4-(1,1,1,3,3,3-hexafluoro-2-hydroxypropan-2-yl)-2,6-dipropylphenyl	CH_3	4-isopropoxyphenyl	H	5.959	6.053	5.921	5.749
33	4-(1,1,1,3,3,3-hexafluoro-2-hydroxypropan-2-yl)-2,6-dipropylphenyl	CH_3	4-(trifluoromethoxy)phenyl	H	5.745	5.740	5.569	5.761
34	4-(1,1,1,3,3,3-hexafluoro-2-hydroxypropan-2-yl)-2,6-dipropylphenyl	CH_3	4-isopropyphenyl	H	5.745	5.813	5.770	5.770
35	4-(1,1,1,3,3,3-hexafluoro-2-hydroxypropan-2-yl)-2,6-dipropylphenyl	CH_3	1,1'-biphenyl-4-yl	H	5.921	5.899	5.796	5.823
36[β,α]	2-oxo-8-propyl-4-(trifluoromethyl)-2H-chromen-7-yl	CH_3	CH_3	CH_3	5.620	5.332	5.991	5.695
37	4-(1,1,1,3,3,3-hexafluoro-2-hydroxypropan-2-yl)-2,6-dipropylphenyl	ethyl	4-methoxyphenyl	H	5.921	5.860	5.959	5.800
38	4-(1,1,1,3,3,3-hexafluoro-2-hydroxypropan-2-yl)-2,6-dipropylphenyl	ethyl	benzo[d][1,3]dioxol-5-yl	H	6.222	6.165	6.155	6.184
39[α]	4-(1,1,1,3,3,3-hexafluoro-2-hydroxypropan-2-yl)-2,6-dipropylphenyl	CH_3	2,3-dihydrobenzo[b][1,4]dioxin-6-yl	H	6.301	6.471	6.222	6.085
40	2-oxo-6,8-dipropyl-4-(trifluoromethyl)-2H-chromen-7-yl	H	H	CH_3	5.585	5.562	5.886	5.962
41[β,α]	2-oxo-6,8-dipropyl-4-(trifluoromethyl)-2H-chromen-7-yl	H	CH_3	CH_3	5.770	5.499	5.886	5.416

42	2-oxo-6,8-dipropyl-4-(trifluoromethyl)-2H-chromen-7-yl	CH_3	CH_3	CH_3	4.959	4.954	5.328	5.374
43	2-oxo-6,8-dipropyl-4-(trifluoromethyl)-2H-chromen-7-yl	CH_3	H	H	5.824	5.858	5.886	5.871
44^{β}	2-oxo-6,8-dipropyl-4-(trifluoromethyl)-2H-chromen-7-yl	CH_3	phenyl	H	ia	ia	5.276	5.295
45	2-oxo-6,8-dipropyl-4-(trifluoromethyl)-2H-chromen-7-yl	CH_3	4-methylphenyl	H	ia	ia	5.620	5.661
46^{β}	2-oxo-6,8-dipropyl-4-(trifluoromethyl)-2H-chromen-7-yl	CH_3	benzo[d][1,3]dioxol-5-yl	H	ia	ia	5.921	5.526
47	2-oxo-6,8-dipropyl-4-(trifluoromethyl)-2H-chromen-7-yl	CH_3	4-nitrophenyl	H	ia	ia	5.097	5.149
48	2-oxo-6,8-dipropyl-4-(trifluoromethyl)-2H-chromen-7-yl	H	H	CH_3	ia	ia	5.886	5.807
49	2-oxo-8-propyl-4-(trifluoromethyl)-2H-chromen-7-yl	CH_3	phenyl	H	ia	ia	5.699	5.816
50^{β}	2-oxo-8-propyl-4-(trifluoromethyl)-2H-chromen-7-yl	CH_3	4-methoxyphenyl	H	ia	ia	5.854	5.477
51	2-oxo-8-propyl-4-(trifluoromethyl)-2H-chromen-7-yl	CH_3	3,4-dimethoxyphenyl	H	ia	ia	5.523	5.472
52	2-oxo-8-propyl-4-(trifluoromethyl)-2H-chromen-7-yl	CH_3	benzo[d][1,3]dioxol-5-yl	H	ia	ia	5.959	6.100
53^{β}	2-oxo-6,8-dipropyl-4-(trifluoromethyl)-2H-chromen-7-yl	CH_3	4-methoxyphenyl	H	ia	ia	6.018	5.684

"$^{\alpha}$" denotes the test set compounds for modeling LXRα agonists; "$^{\beta}$" denotes the test set compounds for modeling LXRβ agonists; nd = not determined; ia = inactive at 10 μM; pEC_{50}^{α} denotes experimental pEC_{50} values for LXRα; pEC_{50}^{β} denotes experimental pEC_{50} values for LXRβ; Predicted pEC_{50}^{α} denotes predicted pEC_{50} values for LXRα by derived LXRα QSAR model; Predicted pEC_{50}^{β} denotes predicted pEC_{50} values for LXRβ by derived LXRβ QSAR model.

Table S2. The correlation matrix of descriptors of LXRβ QSAR model.

	AM1_LUMO	GCUT_SLOGP_2	E_strain	dipoleX	SMR_VSA6	vsurf_DD13	vsurf_IW2	vsurf_IW5	glob
AM1_LUMO	1.000	0.498	-0.123	0.061	-0.192	-0.388	-0.414	0.066	-0.126
GCUT_SLOGP_2	0.498	1.000	0.131	0.200	-0.243	-0.416	-0.467	0.242	0.302
E_strain	-0.123	0.131	1.000	-0.017	-0.032	-0.157	-0.102	-0.191	0.058
dipoleX	0.061	0.200	-0.017	1.000	-0.240	-0.058	-0.130	0.111	0.124
SMR_VSA6	-0.192	-0.243	-0.032	-0.240	1.000	0.177	-0.048	-0.165	0.014
vsurf_DD13	-0.388	-0.416	-0.157	-0.058	0.177	1.000	0.267	-0.309	-0.277
vsurf_IW2	-0.414	-0.467	-0.102	-0.130	-0.048	0.267	1.000	0.239	-0.100
vsurf_IW5	0.066	0.242	-0.191	0.111	-0.165	-0.309	0.239	1.000	0.417
glob	-0.126	0.302	0.058	0.124	0.014	-0.277	-0.100	0.417	1.000

Table S3. The correlation matrix of descriptors of LXRα QSAR model.

	BCUT_SMR_0	GCUT_SLOGP_2	Q_VSA_ FPPOS	Q_VSA_ POS	E_ang	dipoleY	pmiY	SlogP_ VSA2	SlogP_ VSA3	vsurf_CW6	vsurf_DD12	vsurf_ DW12
BCUT_ SMR_0	1.000	0.064	0.192	-0.722	0.082	-0.020	0.051	0.037	-0.094	0.403	-0.135	0.076
GCUT_S LOGP_2	0.064	1.000	-0.266	-0.145	-0.177	-0.100	-0.048	-0.759	0.349	0.514	0.199	0.214
Q_VSA_ FPPOS	0.192	-0.266	1.000	-0.242	0.333	0.295	-0.025	0.303	0.107	-0.042	-0.005	0.125
Q_VSA_ POS	-0.722	-0.145	-0.242	1.000	0.168	0.122	0.087	-0.070	0.099	-0.122	0.050	-0.126
E_ang	0.082	-0.177	0.333	0.168	1.000	0.023	0.232	0.363	-0.151	-0.074	-0.179	-0.176
dipoleY	-0.020	-0.100	0.295	0.122	0.023	1.000	-0.229	0.007	-0.020	-0.043	-0.214	0.348
pmiY	0.051	-0.048	-0.025	0.087	0.232	-0.229	1.000	0.217	0.303	-0.071	0.119	-0.120
SlogP_ VSA2	0.037	-0.759	0.303	-0.070	0.363	0.007	0.217	1.000	-0.183	-0.618	-0.122	-0.324
SlogP_ VSA3	-0.094	0.349	0.107	0.099	-0.151	-0.020	0.303	-0.183	1.000	0.168	0.243	-0.117
vsurf_ CW6	0.403	0.514	-0.042	-0.122	-0.074	-0.043	-0.071	-0.618	0.168	1.000	0.004	0.259
vsurf_ DD12	-0.135	0.199	-0.005	0.050	-0.179	-0.214	0.119	-0.122	0.243	0.004	1.000	-0.269
vsurf_ DW12	0.076	0.214	0.125	-0.126	-0.176	0.348	-0.120	-0.324	-0.117	0.259	-0.269	1.000
